# L2MXception: an improved Xception network for classification of peach diseases

**DOI:** 10.1186/s13007-021-00736-3

**Published:** 2021-04-01

**Authors:** Na Yao, Fuchuan Ni, Ziyan Wang, Jun Luo, Wing-Kin Sung, Chaoxi Luo, Guoliang Li

**Affiliations:** 1 College of Informatics, Huazhong Agricultural University, Wuhan, 430070 Hubei China; 2 Hubei Engineering Technology Research Center of Agricultural Big Data, Wuhan, 430070 Hubei China; 3 College of Information Engineering, Tarim University, Alaer, 843300 Xinjiang China; 4 Department of Computer Science, National University of Singapore, Singapore, 117417 Singapore; 5 Department of Computational and Systems Biology, Genome Institute of Singapore, Singapore, 138672 Singapore; 6 College of Plant Science & Technology, Huazhong Agricultural University, Wuhan, 430070 Hubei China

**Keywords:** Deep learning, Identification, Peach diseases

## Abstract

**Background:**

Peach diseases can cause severe yield reduction and decreased quality for peach production. Rapid and accurate detection and identification of peach diseases is of great importance. Deep learning has been applied to detect peach diseases using imaging data. However, peach disease image data is difficult to collect and samples are imbalance. The popular deep networks perform poor for this issue.

**Results:**

This paper proposed an improved Xception network named as L2MXception which ensembles regularization term of L2-norm and mean. With the peach disease image dataset collected, results on seven mainstream deep learning models were compared in details and an improved loss function was integrated with regularization term L2-norm and mean (L2M Loss). Experiments showed that the Xception model with L2M Loss outperformed the current best method for peach disease prediction. Compared to the original Xception model, the validation accuracy of L2MXception was up to 93.85%, increased by 28.48%.

**Conclusions:**

The proposed L2MXception network may have great potential in early identification of peach diseases.

## Introduction

Peach is an important fruit and its production is affected by peach diseases. The major peach diseases are brown rot, anthracnose, scab, bacterial shot hole, gummosis, powdery mildew, leaf curl, and so on. The diseases deduce the peach production, and thus it is urgently needed to find rapid and accurate methods to identify peach diseases in earlier stage.

There are several ways for diagnosing plant diseases in general and peach diseases in particular. The first way is visual assessment relying on the farmer’s experience; however, it is a subjective task, so that it may cause deviations or even errors. The second way is using spectrometer to diagnose the plant diseases by wavelength [1, 2]; however, the spectrometer cannot be popularized due to its high price. The third way is applying polymerase chain reaction [3–5] by biological operation; however, the experimental procedure is complicated for ordinary farmers. With the development of computer vision, another way is image-based recognition of plant disease, which is proposed and applied widely [6–14]. Ref. [15] proposed a shallow artificial neural network model to analyse images of cherry and plum shoots. These methods use traditional image processing algorithm, and can achieve high performance for a certain type of research objects. However, such computer methods are semiautomatic because different images need different operations, such as the threshold-based segmentation of the lesion areas. Recently, deep learning is rapidly developed and solves the disadvantages of traditional computer vision methods, although it also has its own imperfections, such as relying on a large number of samples. Deep learning has been successfully applied in various fields, such as transportation [16], medical image analysis [17], signal processing [18]. Furthermore, the deep learning is also used in agriculture, such as weed identification [19], plant identification [20], pest identification [21], and plant disease detection [22–26]. Nagasubramanian, [27] demonstrated that a 3D CNN model can be used effectively to learn from hyperspectral data to identify charcoal rot disease in soybean stems. Especially, Zhang et al. [28] compared deep learning and traditional methods in identification of peach leaf disease infected by Xanthomonas campestris, drawing a conclusion that convolutional neural network is significantly superior than the traditional methods, such as Support Vector Machine, Nearest Neighbor and Back Propagation neural network.

**Fig. 1:**
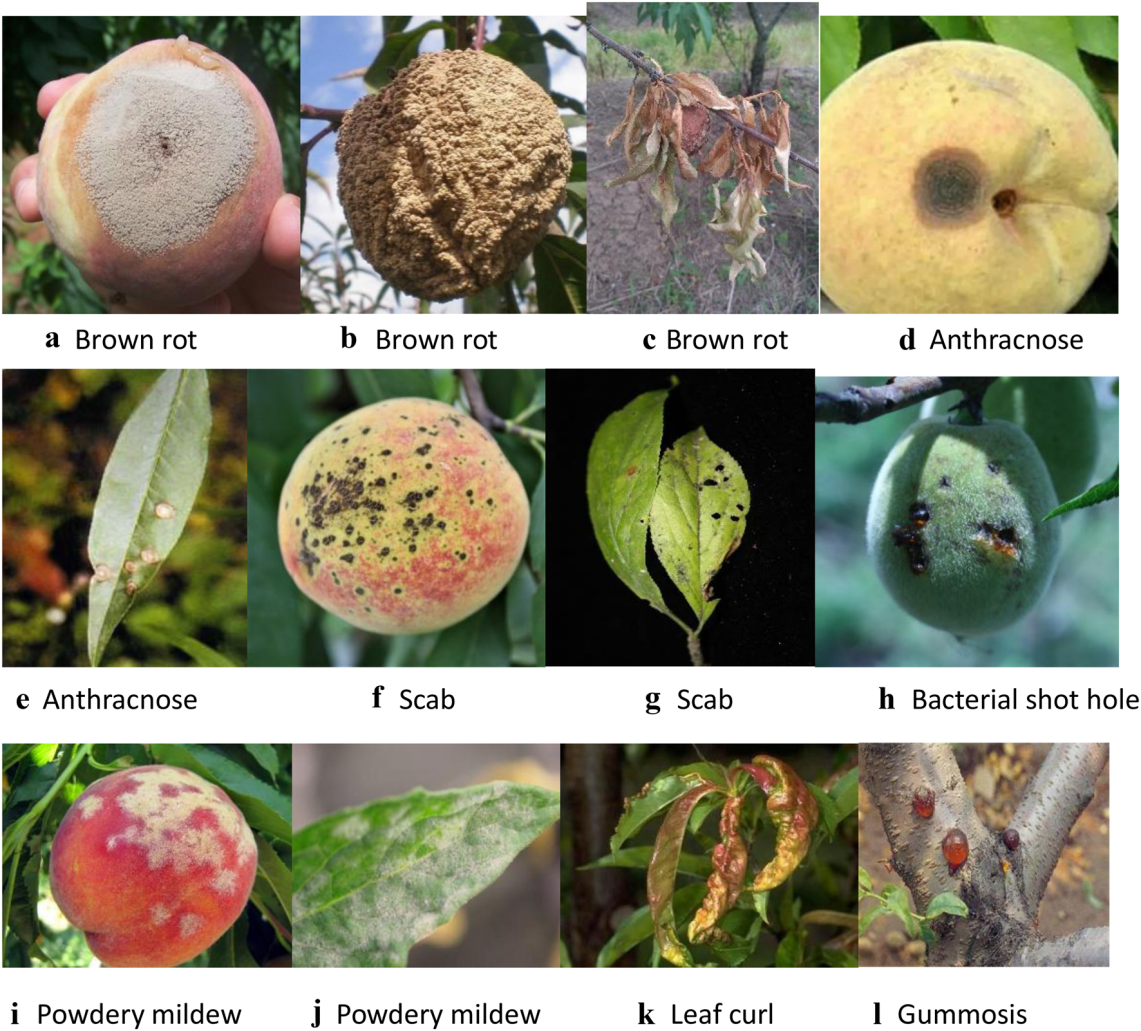
Major plant diseases of peach. **a** Brown rot for fruit. **b** Brown rot for fruit. **c **Brown rot for leaf. **d** Anthrax for fruit. **e** Anthrax for leaf. **f** Scab for fruit. **g** Scab for leaf. **h** Bacterial perforation for fruit. **i** Powdery mildew for fruit. **j **Powdery mildew for leaf. **k** Leaf curl for leaf. **l** Gummosis for branch

In this paper, we focus on the identification of 7 major peach diseases (brown rot, anthracnose, scab, bacterial shot hole, gummosis, powdery mildew, leaf curl, as shown in Fig. 1) with deep convolutional neural networks (CNN) Models. The peach disease image dataset, was collected *from peach orchards by Prof. Luo’s team, College of Plant Science and Technology, HZAU*, which includes 7 categories of peach disease images. The 7 categories are 1) Brown rot fungi infecting fruits and leaves, 2) Anthracnose fungi infecting fruits and leaves, 3) Scab fungus infecting fruits, branches and leaves, 4) Shot hole bacterium infecting fruits, branches and leaves, 5) Gummosis fungi infecting branches, 6) Powdery mildew fungus infecting fruits and leaves and 7) Leaf curl fungus infecting leaves. These diseases bring damages to different parts of the peach plant (see Fig. 1). For example, the brown rot disease mainly harms the fruit, causing the fruit to rot, which also harms the leaves and causes the leaves to dry up. Gummosis mainly harms branches, causing tree weakness, affecting fruit quality, and even causing death of branches and trees. The 7 diseases were researched in the laboratory, so laboratory personnel were familiar with the characteristics of the diseases. For example, a certain disease mainly infects fruits, and leaves and branches are also infected a few, so the disease images were mainly collected by fruit pictures. The project team is a team of experts on fruit disease prevention and control posts in the National Peach Industry Technology System, which can ensure the accuracy of its classification. For similar diseases and diseases that are easy to be confused, accurate conclusions can be drawn through tissue isolation of pathogenic bacteria or direct monospore isolation, pathogen morphology observation and molecular biological identification. The collection methods were two ways. The first way was collecting pictures of existing resources in the laboratory or obtaining some pictures from other experts through cooperation in the Peach system, and the second way was taking a large number of pictures indoors or orchards.

Comparing with seven existing deep CNN models, the results showed that DenseNet169 had the highest validation accuracy (89.32%). In order to improve accuracy, by analyzing data distribution of peach disease image dataset and the results based on seven existing deep learning models, we proposed to apply regularization to seven existing models. The Xception model with regularization term of L2-norm achieved the highest validation accuracy of 92.23%. Furthermore, when regularization term was changed to L2-norm and mean, the validation accuracy was further improved to 93.85%.

## Result and discussion

**Fig. 2:**
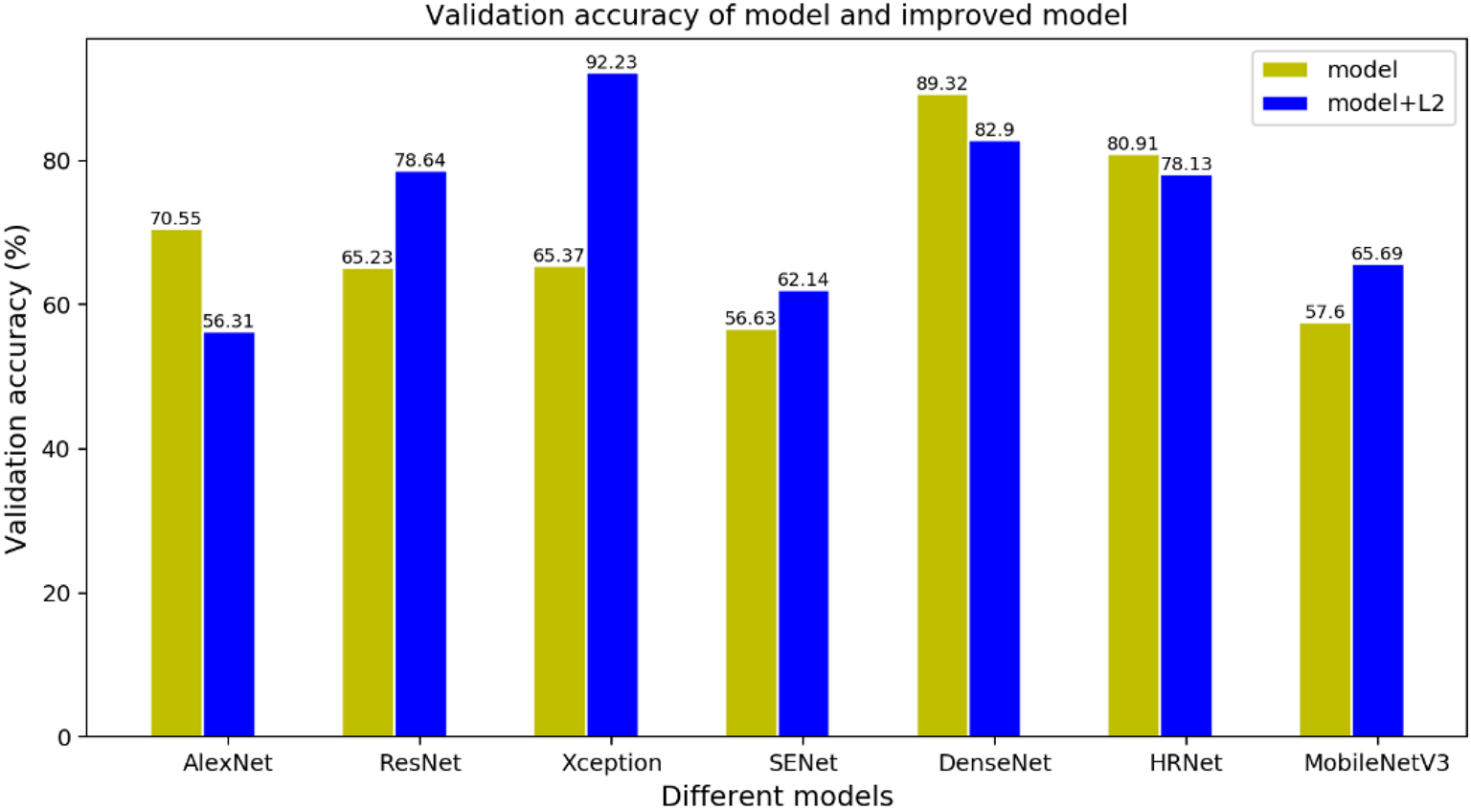
Validation accuracies of seven models and seven improved models

The results presented in Fig. 2 show that the models applying regularization with L2-norm achieved better performance compared to original CNN models except AlexNet, DenseNet and HRNet.

For the original models, DenseNet had the highest validation accuracy of 89.32% and SENet had the lowest validation accuracy of 56.63% as shown in Table 1.

**Table 1:** Results and parameters based on seven original models

Network	Batch size	Epoch	Learning rate	Training accuracy (%)	Validation accuracy (%)
AlexNet	64	60	0.001	72.02	70.55
ResNet50	64	60	0.001	68.28	65.23
Xception	64	60	0.001	67.86	65.37
SENet154	64	60	0.001	53.00	56.63
DenseNet169	32	60	0.001	90.49	89.32
HRNet-w48	64	60	0.001	89.06	80.91
MobileNetV3	64	60	0.001	57.63	57.60

When there are many predictors in the dataset and not all of predictors have the same predicting power, L2-norm regularization can be used to estimate the predictor importance and penalize predictors that are not important. When the L2-norm regularization is added to the loss function, overfitting problem will be solved better. For the methods with L2-norm regularization, validation accuracy increased by 26.86%, 13.41%, 8.09% and 5.51% for Xception, ResNet, MobileNetV3 and SENet, respectively. However, the validation accuracy decreased by 14.24%, 2.78% and 6.42% for AlexNet, HRNet and DenseNet, respectively. The validation accuracy of DenseNet and HRNet were slightly reduced after L2-norm regularization. The highest validation accuracy was 92.23% for Xception after applying regularization with L2-norm.

**Fig. 3:**
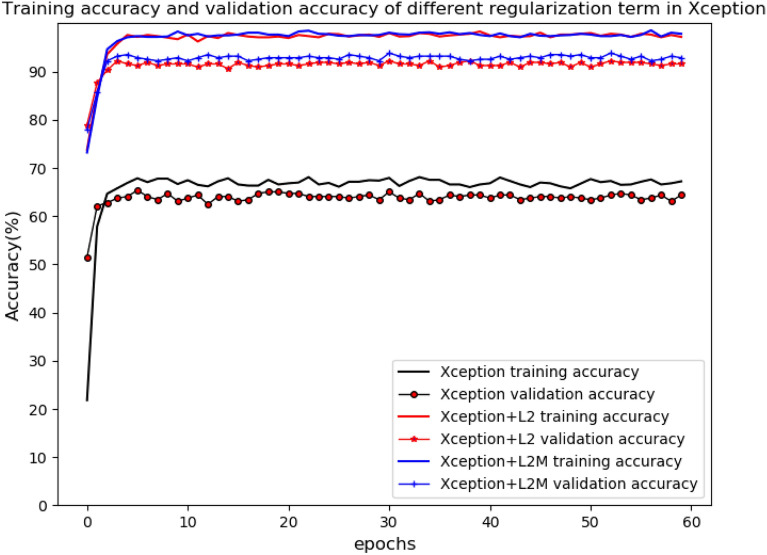
Training accuracy and validation accuracy in the original Xception and the Xception with different regularization term

**Fig. 4:**
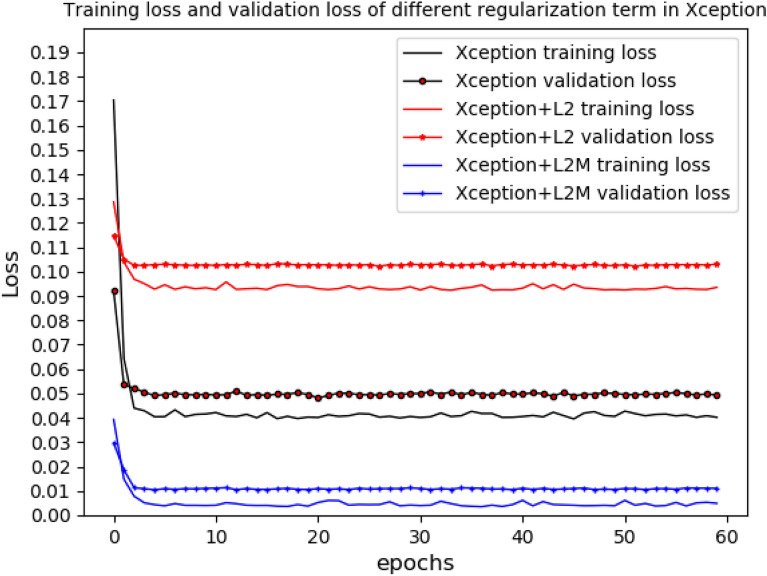
Training loss and validation loss in the original Xception and the Xception with different regularization term

**Fig. 5:**
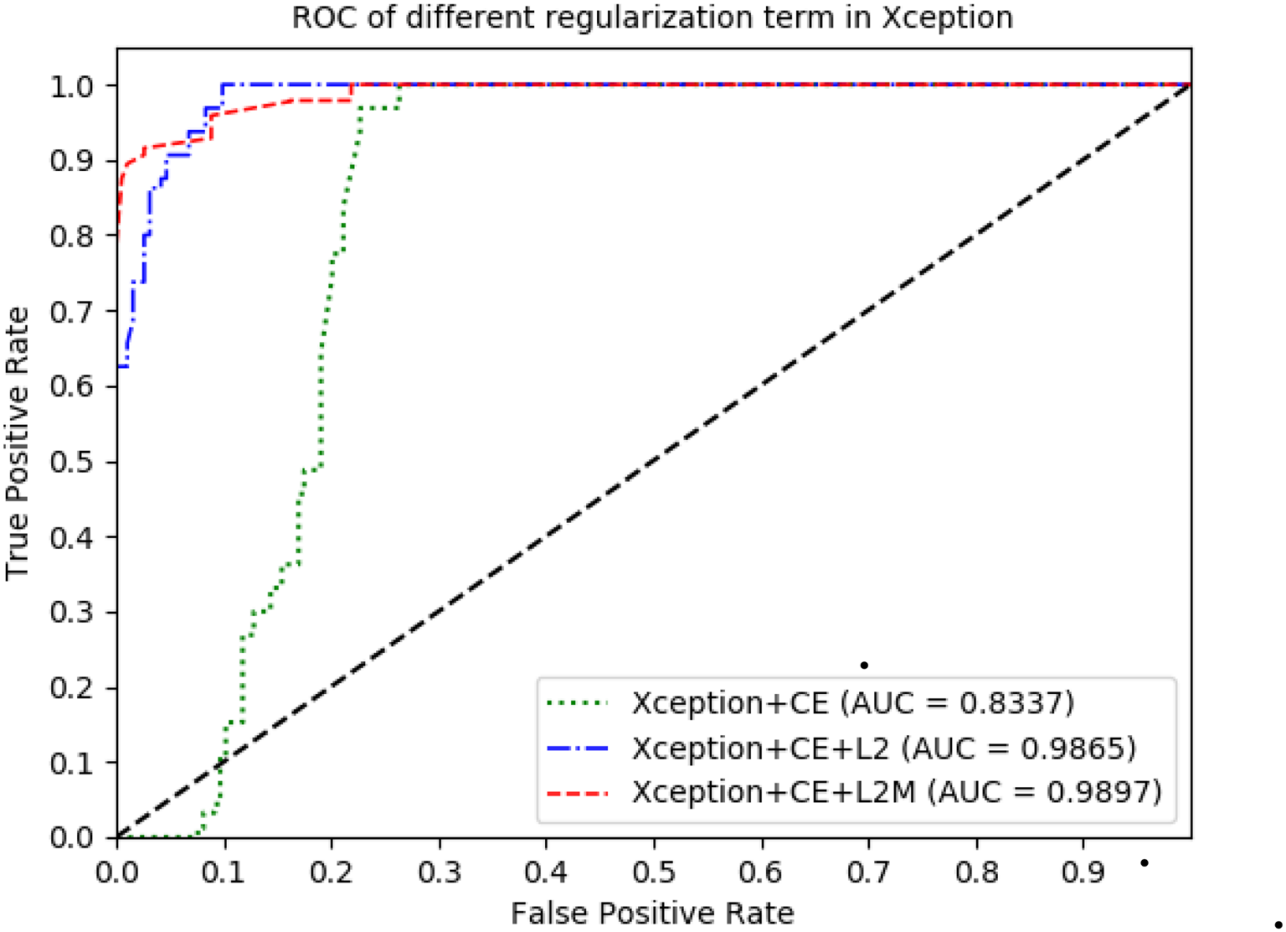
ROC of the original Xception and the Xception with different regularization term

Regularization with L2-norm was most effective for Xception. On the basis of L2-norm, in order to improve the model the regularization term $\lambda \parallel w{\parallel }_{2}$ in Eq. (2) was changed to two parts of ${\gamma }_{1}\frac{1}{N}{\sum }_{i=0}^{N-1}{w}_{i}+{\gamma }_{2}{\Vert w\Vert }_{2}$ as shown in Expression (3) consequently. After testing different parameter values of ${\gamma }_{1}$ and ${\gamma }_{2}$ as shown in Table 2, we found that when ${\gamma }_{1}\,=\,0.7$ and${\gamma }_{2}\,=\,0.3$, the validation accuracy of Xception was up to 93.85% as shown in Table 2. The parameters of $\gamma_{{1}} ,\gamma_{2}$ are chosen to suitable value for better performance. Thus, it can be seen that regularization can make the performance of Xception better. The training accuracy and validation accuracy in the original Xception and Xception with different regularization term was shown in Fig. 3. The training accuracy was average accuracy per epoch, and so was the validation accuracy. The results also showed that regularization for Xception can greatly improve training accuracy and validation accuracy. The training accuracy of Xception with L2-norm is not much different from that of Xception with L2-norm and mean, but the validation accuracy of Xception with L2-norm and mean was obviously higher than that of Xception with L2-norm. Furthermore, training loss and validation loss in the original Xception and Xception with different regularization term was shown in Fig. 4. Receiver operating characteristic (ROC) of the original Xception and Xception with different regularization term was shown in Fig. 5, which also showed area under curve (AUC) of the original Xception and Xception with different regularization term in the legend at the bottom right. The AUC of L2MXception model outperformed the other two methods.

**Table 2:** Different results corresponding to different parameters based on Xception(L2 and mean)

Parameters value (${\gamma }_{1}{,}\,{\gamma }_{2}$)	Validation accuracy (%)
${\gamma }_{1}=0{,}\,{\gamma }_{2}=1$	92.23
${\gamma }_{1}= {0.5} {,}\,{\gamma }_{2}={0.5}$	92.88
${\gamma }_{1}={0.6}{,}\,{\gamma }_{2}={0.4}$	91.64
${\gamma }_{1}={0.7}{,}\,{\gamma }_{2}={0.3}$	93.85
${\gamma }_{1}={0.8}{,}\,{\gamma }_{2}={0.2}$	92.88
${\gamma }_{1}=1{,}\,{\gamma }_{2}=0$	92.56
${\gamma }_{1}=0{,}\,{\gamma }_{2}=0$	65.37

When regularization with L2-norm and mean(L2M) was used in seven models, the validation accuracy was shown in Table 3. Training parameters (epoch, learning rate and batch size) of seven models are same in Table 1 and the value of $\gamma_{{1}}$ and $\gamma_{{2}}$ were ${\gamma }_{1}={0.7}{,}\,{\gamma }_{2}={0.3}$. The Xception with L2 and L2M both can improved the validation accuracy, while the Xception with L2M improved less compared to Xception with L2. The regularization with L2 and L2M were not suitable for all seven models, as shown in Table 3 and Fig. 2, DenseNet169, HRNet-w48 and AlexNet were not suitable for using regularization with L2 and L2M. Maybe using regularization with L2 and L2M is repeated for DenseNet169, because the network includes actions for preventing overfitting. HRNet-w48 is more complex than ResNet50. Also AlexNet is complex and it’s pre-trained model is lager than other four models. Maybe according to the reasons, the regularization with L2 and L2M are not suitable for them.

**Table 3:** The comparison of Validation accuracy of seven models with L2 and L2M

Network	Validation accuracy(L2) (%)	Validation accuracy (L2M) (%)	Change
AlexNet	56.31	57.31	1.00% (+)
ResNet50	78.64	79.34	0.7% (+)
Xception	92.23	93.85	1.62% (+)
SENet154	62.14	62.84	0.7% (+)
DenseNet169	82.90	80.58	2.32% (−)
HRNet-w48	78.13	78.00	0.13% (−)
MobileNetV3	65.69	66.01	0.32% (+)

We also experimented this dataset using Xception with regularization of L1-norm and L2-norm, and the validation accuracy was shown in Table 4. In this case, the regularization term $\lambda \parallel w{\parallel }_{2}$ in Eq. (2) was changed to $\gamma_{{3}} \left\| w \right\|_{1} + \gamma_{4} \left\| w \right\|_{2}$, and the loss function is Eq. (5). The parameters of $\gamma_{{3}} ,\gamma_{4}$ are chosen to suitable value for better performance. The results in Table 3 showed that regularization with L2-norm and mean was better than regularization with L1-norm and L2-norm based on Xception.

**Table 4:** Different results corresponding to different parameters based on Xception (L1 and L2)

Parameters value ($\gamma_{3}{,}\, \gamma_{4}$)	Validation accuracy (%)
${\gamma }_{3}=0{,}\,{\gamma }_{4}=1$	92.23
${\gamma }_{3}={0.5}{,}\,{\gamma }_{4}={0.5}$	88.35
${\gamma }_{3}={0.6}{,}\,{\gamma }_{4}={0.4}$	87.06
${\gamma }_{3}={0.7}{,}\,{\gamma }_{4}={0.3}$	87.70
${\gamma }_{3}={0.8}{,}\,{\gamma }_{4}={0.2}$	86.41
${\gamma }_{3}=1{,}\,{\gamma }_{4}=0$	86.73
${\gamma }_{3}=0{,}\,{\gamma }_{4}=0$	65.37

**Fig. 6:**
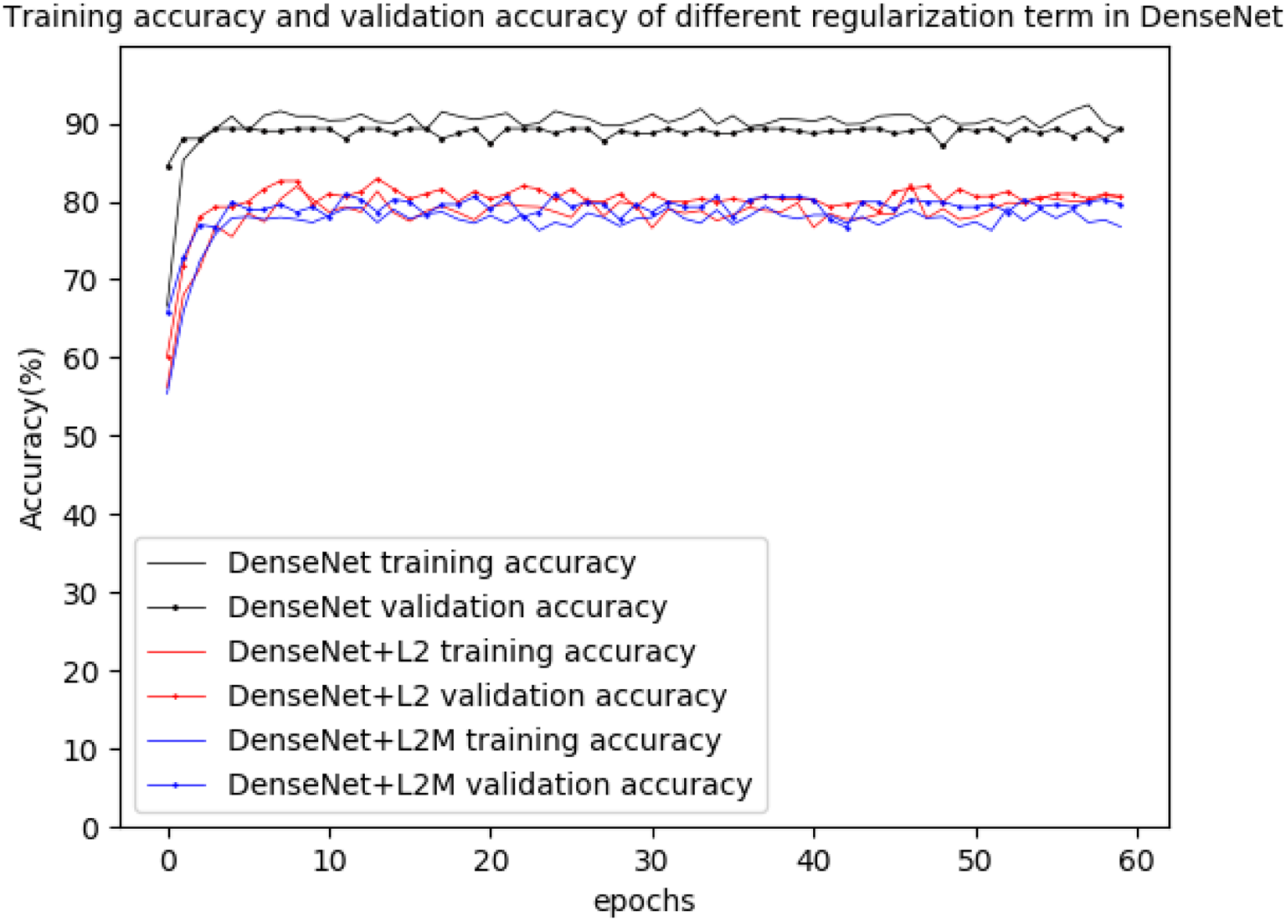
Training accuracy and validation accuracy in the original DenseNet169 and the DenseNet169 with different regularization term

**Fig. 7:**
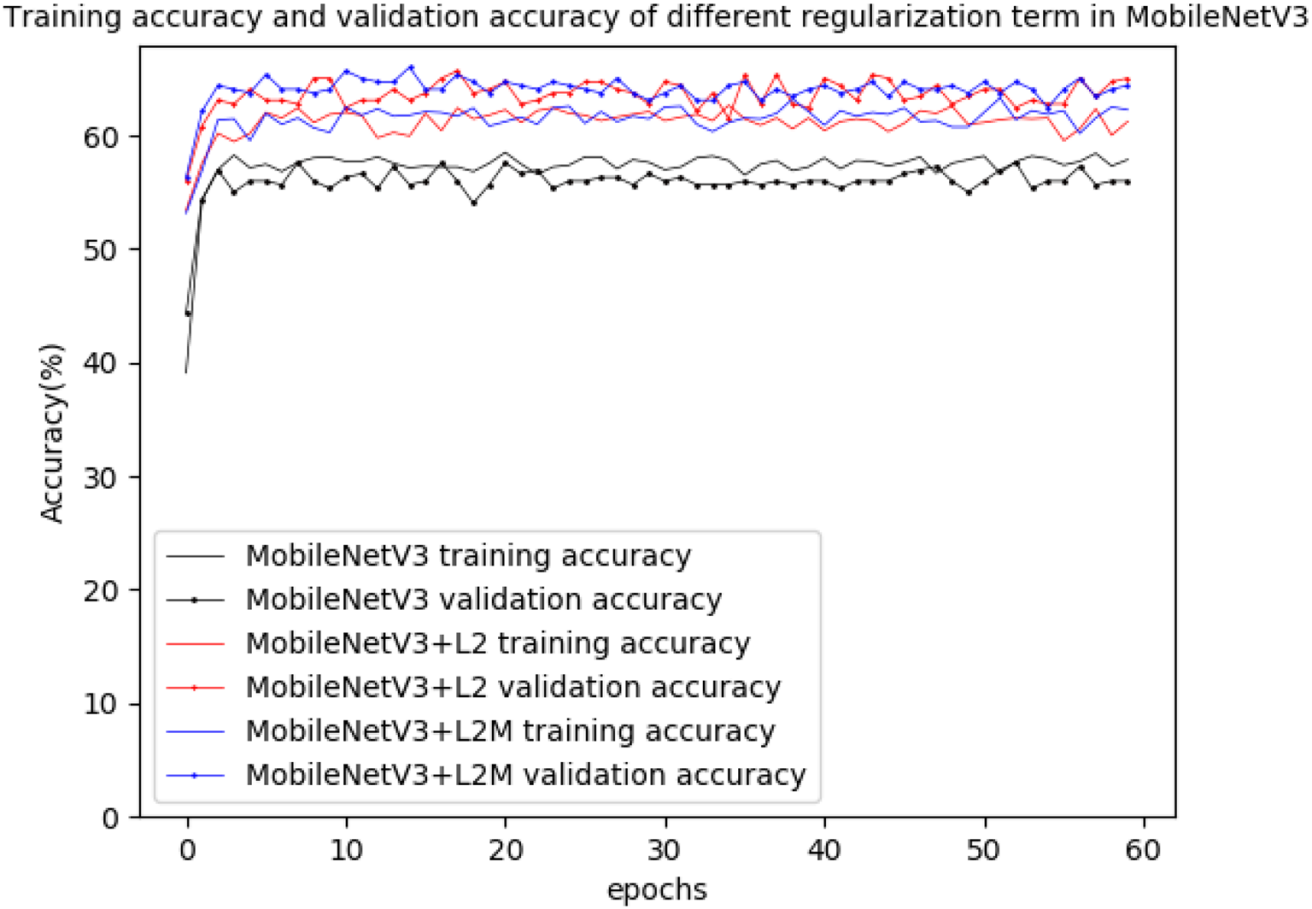
Training accuracy and validation accuracy in the original MobileNetV3 and the MobileNetV3 with different regularization term

**Fig. 8:**
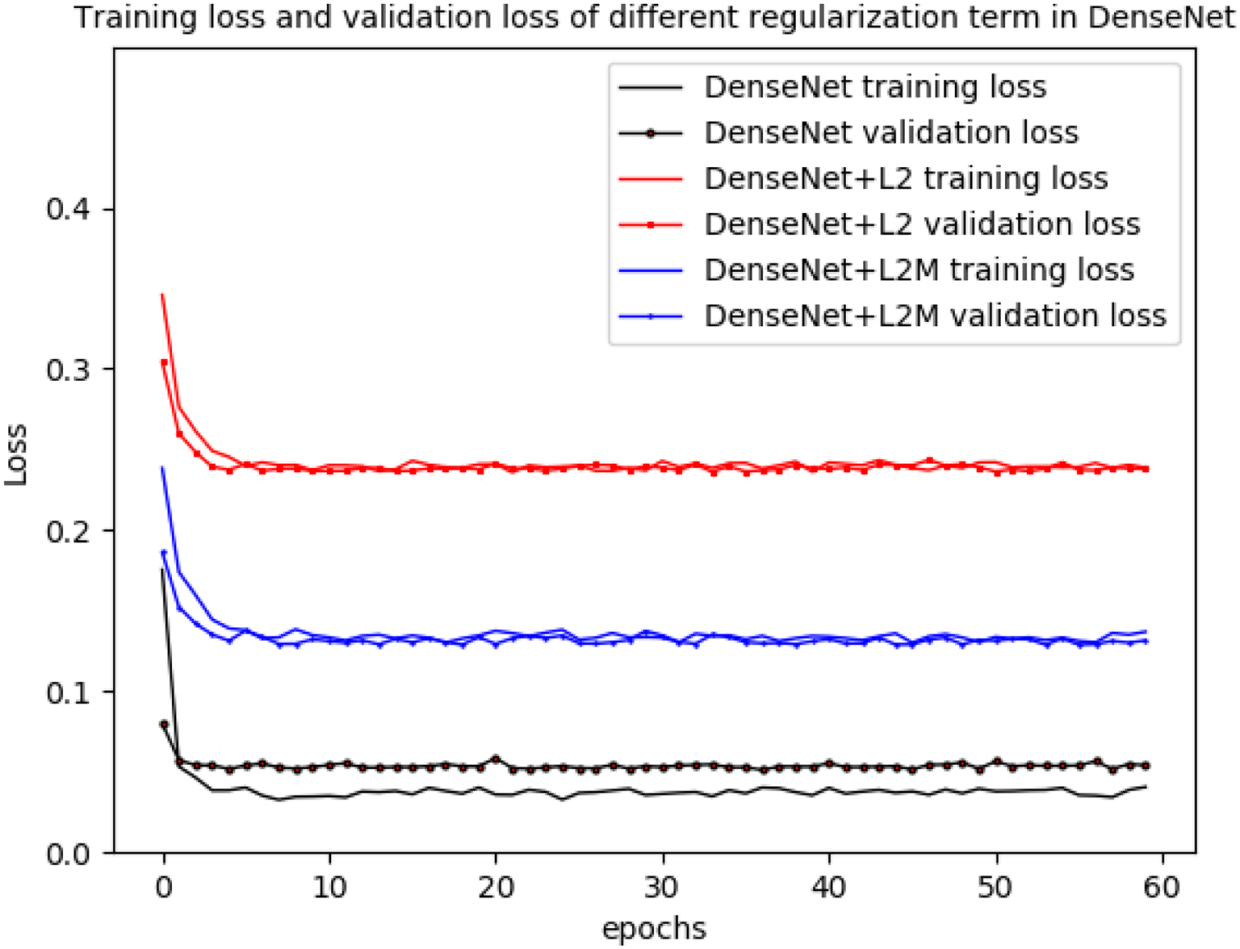
Training loss and validation loss in the original DenseNet169 and the DenseNet169 with different regularization term

**Fig. 9:**
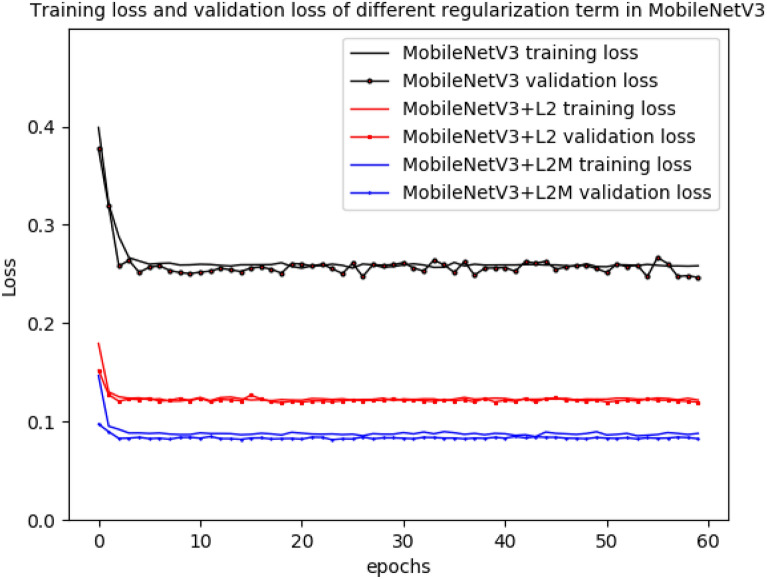
Training loss and validation loss in the original MobileNetV3 and the MobileNetV3 with different regularization term

The accuracy of DenseNet169 and MobileNetV3 was shown in Figs. 6 and 7, while the loss of DenseNet169 and MobileNetV3 was shown in Figs. 8 and 9.

We used test dataset on Xception, Xception with L2 and Xception with L2M, and the test accuracy is 64.32%, 91.67% and 92.16%, respectively.

## Conclusions

In this paper, an improved Xception Network ensemble with L2M Loss was proposed for classification of peach diseases. And seven deep learning models were applied to identify peach diseases from images. The disease image dataset has 7 kinds of diseases and 1560 images, including infected different parts such as fruits, branches and leaves. In the dataset 1251 images are used for train and 156 images are used for validation and 153 images are used for test. The highest validation accuracy was 89.32% based on original DenseNet169 model. By analyzing the data distribution and classification results of seven deep learning models, the improved methods with regularization were proposed to improve accuracy. After experiments, the highest validation accuracy is 93.85% from Xception model with regularization term of L2-norm and mean. But the regularization with L2 and L2M were not effective for all seven models, and regularization with L2 and L2M for DenseNet169, HRNet-w48 and AlexNet were not effective. Because the DenseNet169 network includes actions for preventing overfitting, so regularization with L2 and L2M is excess. HRNet-w48 is based on ResNet50, but it’s more complex than ResNet50. Also AlexNet’s pre-trained model is lager than other four models. Maybe according to the reasons, the regularization with L2 and L2M are not effective for them.

ResNet50, Xception, SENet154 and MobileNetV3 get higher validation accuracy by using regularization with L2 and L2M. The experiments show that regularization is highly suitable for Xception model. Furthermore, when regularization term was changed to L2M loss from L2 loss, the validation accuracy was up to 93.85% based on Xception. The proposed method can help to identify peach plant diseases in earlier stage, rapidly and accurately. We will tailor the improved Xception network into Intelligent embedded system in the future.

## Methods

### Peach disease image dataset

**Fig. 10:**
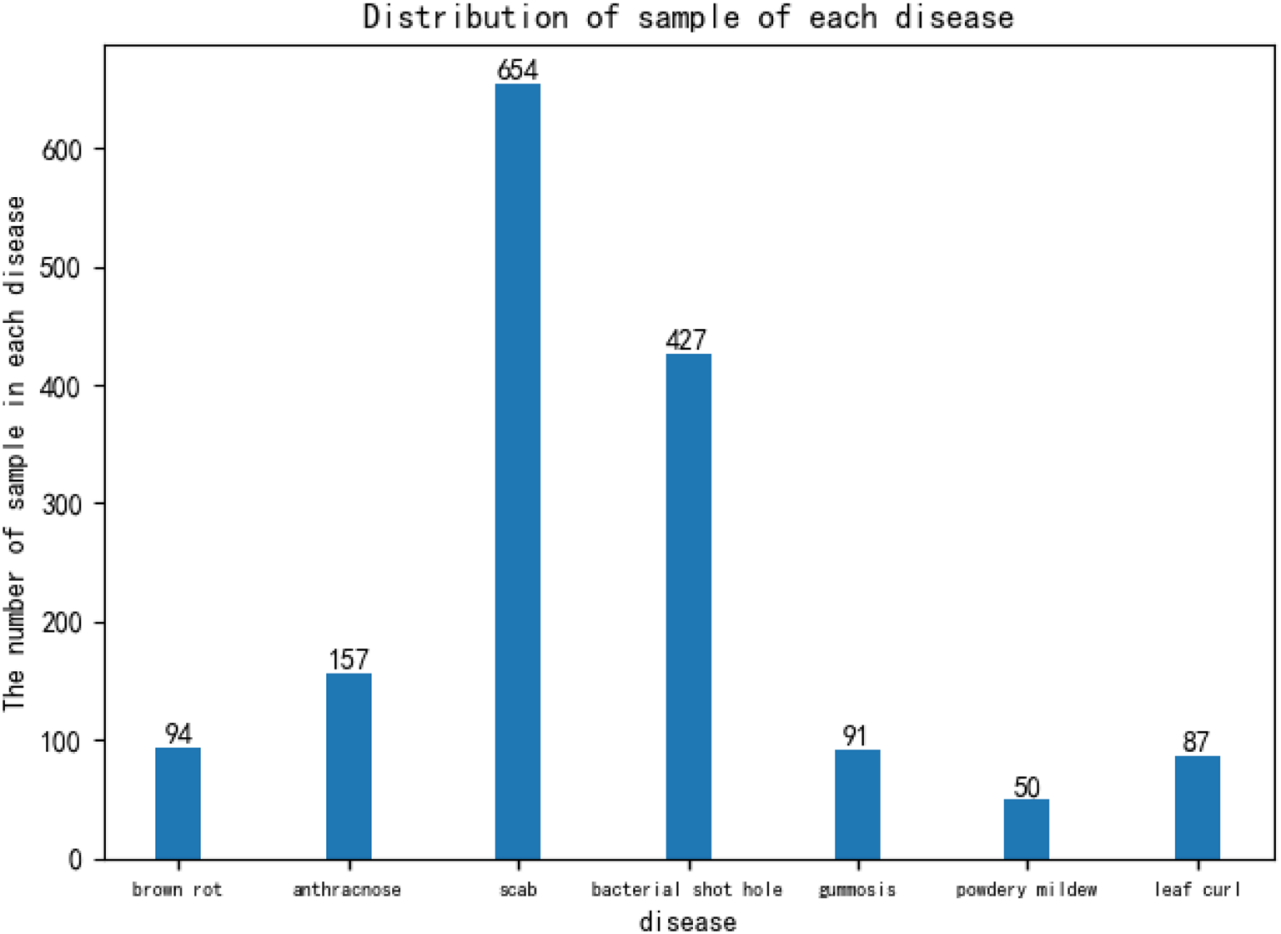
Distribution of sample of each disease

The images of peach diseases were formed into the Peach Disease Image Dataset (PDID). the numbers of each categories in PDID are shown in Fig. 10. The numbers of images of brown rot disease, anthracnose disease, scab disease, bacterial shot hole disease, gummosis disease, powdery mildew disease and leaf curl disease are 94, 157, 654, 427, 91, 50 and 87, respectively. Figure 10 shows that the distribution of the numbers of images of different disease classes are extremely imbalanced. The numbers of images of training dataset, validating dataset, and testing dataset are 1251, 156, and 153, respectively.

### Convolutional neural network

Convolutional neural network (CNN) has become one of the research hotspots in the field of pattern classification. Since the method avoids the complicated pre-processing of images, CNN can directly deal with the original images, and extract features automatically. Convolutional neural networks are very similar to ordinary back-propagation neural networks, and they all consist of neurons with learnable weights and constant bias. Each neuron receives inputs and make mathematical calculations. When $x_{i}$ as inputs, the output of single neural network is: $output = f(\sum\limits_{i = 1}^{n} {w_{i} } x_{i} + b)$. Where $w_{i}$ is weight and $b$ is constant bias. The convolutional neural network output is the score of each classification. The default input of convolutional neural network is an image that allows us to encode specific properties into the network structure, making the feedforward functions more efficient and reducing a large number of parameters.

The basic structure of CNN is composed of convolutional layer, rectified linear units layer, pooling layer and fully connected layer. Each convolutional layer consists of several convolutional units, and the parameters of each convolutional unit are optimized by a backpropagation algorithm. The convolution operation is to extract different features of the input. The first layer of convolutional layer may only extract some low-level features such as edges, lines and corners. The following layers can iteratively extract more complex features from low-level features. The Rectified Linear units (ReLU) layers mainly perform a nonlinear mapping on the output of the convolutional layer. The excitation function used in this layer is generally a ReLU function: $ReLU(x)=\mathit{max}(0,x)$. The pool layers reduce the dimension of each feature map, and the depth of the output remains the same as the number of feature maps. The fully connected layers combine all the local features into global features to calculate the score for each class lastly.

**Fig. 11:**
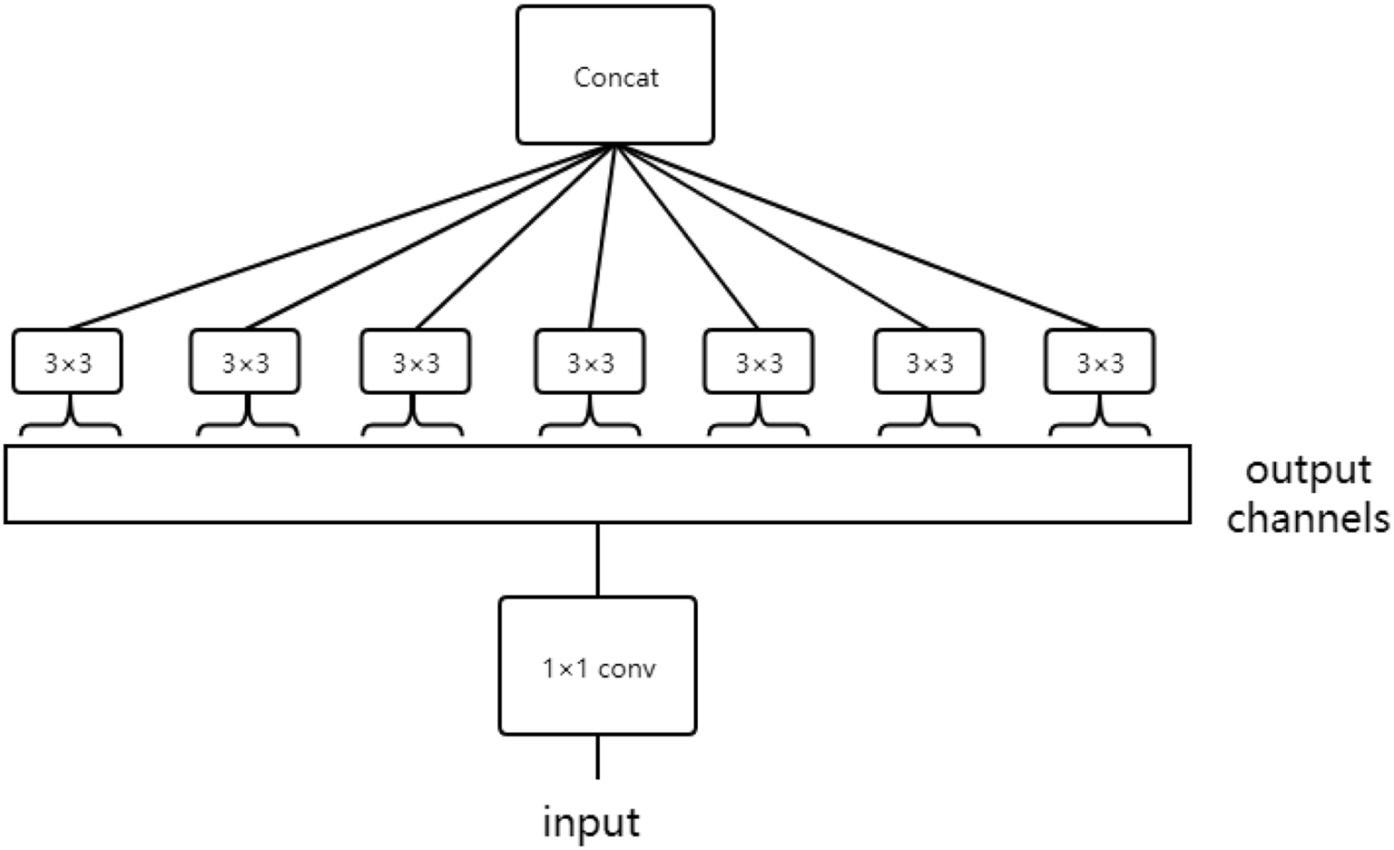
Depthwise separable convolution

**Fig. 12:**
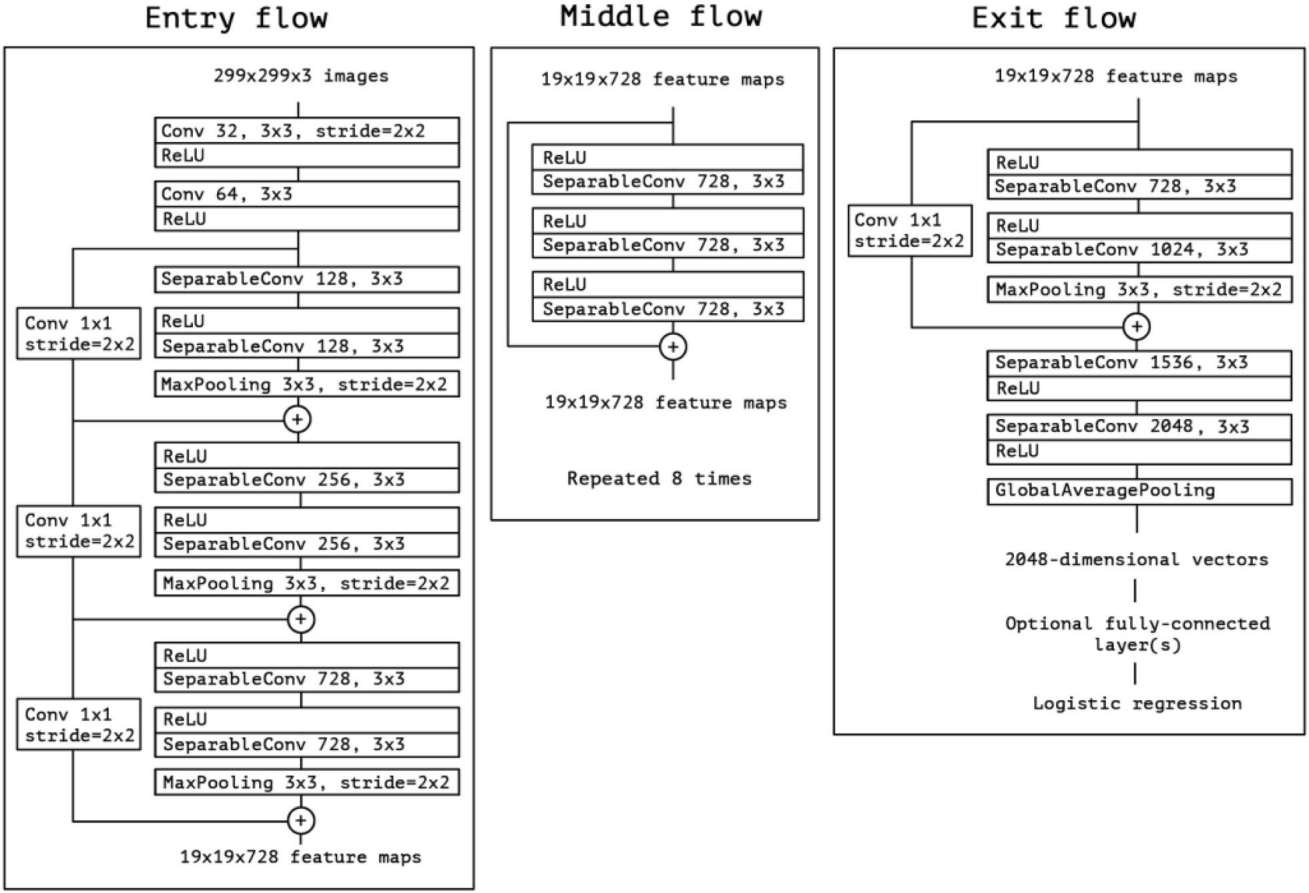
The Xception architecture

CNN was proposed in LeNet network [29] with four typical layers. The AlexNet [30] detonates the application boom of convolutional neural networks, which was the champion of the Large Scale Visual Recognition Challenge 2012 (ILSVRC2012). Since then, more deeper convolutional neural networks are proposed, such as VGG (Simonyan K and Zisserman A, 2014) [31], GoogLeNet [32], ResNet [33], Xception [34], SENet [35], DenseNet [36], HRNet [37], MobileNetV3 [38] and so on. GoogLeNet was the champion of the ILSVRC-2014 competition. The VGG describes that the depth of the network is the key factor for the performance of the algorithm and performs better than GoogLeNet in some Transfer Learning tasks. The ResNet proposes the idea of residual learning and many later models are designed on ResNet network. The structure of Xception is based on ResNet, but the convolutional layer is replaced by depthwise separable convolution as shown in Fig. 11. Although separable convolution can bring about an increase in accuracy or a significant drop in theoretical calculations, due to the scattered calculation process, the efficiency is not high enough. Complete description of the Xception network is presented in the Chollet and François’s paper [34] and the Xception architecture [34] is shown in Fig. 12. Owning to feature reuse and setting bypassing, the parameter amount of DenseNet network is greatly reduced, and the problem of the gradient vanishing is alleviated, while the network has a certain effect of regularization. HRNet, based on residual unit, connects high-to-low resolution convolutions in parallel, where there are repeated multi-scale fusions across parallel convolutions. MobileNetV3 is a combination of depthwise separable convolutions, inverted residual with linear bottleneck and the light weight attention model. AlexNet, ResNet, Xception, SENet, DenseNet and HRNet were applied for classifying peach diseases in this paper. Transfer learning was used to initialize weights for AlexNet, ResNet, Xception, SENet and DenseNet, while HRNet and MobileNetV3 were trained directly by the peach disease image dataset. The pretrained models of AlexNet [39], ResNet [40], Xception [41], SENet [42] and DenseNet [43]are provided by pytorch.

### Image preprocessing

The samples in the dataset are RGB images. Generally, deep learning models have four image preprocessing steps. Images were processed as following stages: firstly, all the images were resized to 224 $\times$ 224 pixels for AlexNet, ResNet50, SEnet, DenseNet, MobileNetV3, 299 $\times$ 299 for Xception, and 256 $\times$ 256 for HRNet. Model optimization and prediction were performed on the rescaled images. Secondly, all pixel values were divided by 255 to [0.0, 1.0]. Thirdly, Z-Score normalization was performed, which was carried out as follows: for each pixel value $x$ as input, mean value ${m}_{x}$ and standard deviation ${s}_{x}$ were calculated and then input $x$ is turned to ${x}{^{\prime}}\,=\,x-{m}_{x}/{s}_{x}$, so that the normalized data was a standard normal distribution with zero mean and unit variance. Finally, several augmentations including random rotation (10), cropping, and flipping (0.5) were used on the training, validating and testing dataset. Rotation, cropping and flipping are random. The parameters of affine transformation for training is degree (−10,10), translate (0.15,0.15), scale (0.9,1.1) and shear (10). Degree (−10,10) represents the range of rotation degree is (−10, 10); Translate(0.15,0.15) represents horizontal shift is randomly sampled in the range (image_width × 0.15, image_width × 0.15) and vertical shift is randomly sampled in the range (image_height × 0.15, image_height × 0.15); Shear (10) represents a shear parallel to the x axis in the range (−10,10) will be applied. The augmentation was helpful for enhancing generalization ability of model and preventing overfitting.

### Regularization to improve CNN models

This paper applied seven CNN models (AlexNet, ResNet, Xception, SENet, DenseNet, HRNet and MobileNetV3) for classifying peach disease images. The parameters and prediction accuracies of all models are shown in Table 1. The best validation accuracy was 89.32% in DenseNet169 and the lowest validation accuracy was 56.63% in SENet. Samples in this dataset were imbalanced, and the number of samples was relatively small. So, too simple model may not work well for this dataset.

In addition to the loss function of CrossEntropyLoss, an additional term is added which varies depending on L1-norm, L2-norm or other combination terms. This additional term is called regularization term which helps to avoid overfitting (L2) and perform features selection (L1). The total loss function with regularization term:

1 $$loss = CrossEntropyLoss + \lambda (regularization \, term)$$

Here, if $\lambda$ is zero then we get back CrossEntropyLoss. However, if $\lambda$ is very large then it will add too much weight and it will lead to under-fitting. So, when $\lambda$ is chosen to a suitable value, this technique works well. In Eq. (1), if regularization term is L1, L1 is $\left\| w \right\|_{1} = \sum\limits_{i = 1} {\left| {w_{i} } \right|}$; if regularization term is L2, L2 is $\left\| w \right\|_{2} = \sqrt {\sum\limits_{i = 1} {\left| {w_{i} } \right|}^{2} }$; The CrossEntropyLoss(CE) is:

$CrossEntropyLoss = \frac{1}{N}\sum\limits_{i} {L_{i} } = \frac{1}{N}\sum\limits_{i} { - \sum\limits_{c = 1}^{M} {y_{ic} \log (p_{ic} )} }$. Where $N$ is the number of samples; $M$ is the number of categories; If the category is the same as the category of sample i, $y_{ic}$ is 1, otherwise it is 0; $p_{ic}$ is the predicted probability that the observed sample i belongs to category c.

To avoid overfitting due to imbalanced samples when training the models, we devised regularization term with L2 to the loss function and the loss has two parts:

2 $$loss = CE + \lambda \left\| w \right\|_{2}$$

where $\lambda$ is a weight decay constant that controls the balance between better fitting of the training data using the term CrossEntropyLoss and minimizing the parameter($w$) values using the regularization term $\lambda \parallel w{\parallel }_{2}$. To further improve the model, we add a term of mean in the regularization term and replace $\lambda \parallel w{\parallel }_{2}$ by two terms:

3 $${\gamma }_{1}\frac{1}{N}{\sum }_{i=0}^{N-1}{w}_{i}+{\gamma }_{2}{\Vert w\Vert }_{2}$$

where ${\gamma }_{1}$ and ${\gamma }_{2}$ are constant coefficients for the first term and the second term, $\frac{1}{N}{\sum }_{i=0}^{N-1}{w}_{i}$ is the mean of $w$.

In total, our L2M loss function is:

4 $$loss = CE + \gamma_{{1}} \frac{1}{N}\sum\nolimits_{i = 0}^{N - 1} {w_{i} } + \gamma_{{2}} \left\| w \right\|_{2}$$

Based on experiments, when ${\gamma }_{1}=0.7$ and ${\gamma }_{2}=0.3$, the validation accuracy of L2MXception network is up to 93.85%. (Shown as Table 2.)

We also do some experiments when the regularization terms conclude L1 and L2:

5 $$loss = CE + \gamma_{{3}} \left\| w \right\|_{1} + \gamma_{{4}} \left\| w \right\|_{2}$$

When $\gamma_{{3}} \gamma_{{4}}$ has the same values with $\gamma_{{1}} \gamma_{{2}}$ respectively, the validation accuracy of Xception network with loss function in Eq. (5) is lower than the validation accuracy of L2MXception network with loss function in Eq. (4).

### Implementation

The experiment of classification was performed on a CentOS workstation equipped with two Intel(R) Xeon(R) E5-2683 v4 CPU (55G RAM), accelerated by two Tesla P100-PCIE GPU (16 GB memory). The model implementation in this paper was powered by deep learning framework of PyTorch.

All applied CNN models in this paper were trained using parameters shown in Table 1. All CNN models used the same training parameters (epoch, learning rate and batch size) except DenseNet169 because of using more memory. These parameters gave the best results during training after appropriate experimentation.

Running time per epoch of different network is shown in Table 5. This running time is an average time of 60 epochs.

**Table 5:** Running time per epoch

network	Original(Second)	Original + L2 (Second)	Original + L2M(Second)
AlexNet	4.05	5.36	7.23
ResNet50	13.53	14.64	22.26
Xception	18.12	18.59	27.6
SENet154	45.86	54.76	78.59
DenseNet169	19.63	28.14	46.24
HRNet-w48	2.26	2.14	2.26
MobileNetV3	4.24	4.96	7.58

## Data Availability

The dataset used and/or analyzed during the current study available from the corresponding author on reasonable request.
